# Tetramethylpyrazine attenuates the blood-brain barrier damage against ischemic stroke by targeting endothelin-1/Akt pathway in astrocytes

**DOI:** 10.3389/fphar.2025.1571552

**Published:** 2025-05-16

**Authors:** Minzhen Deng, Yuefang Cai, Yu Wang, Dafeng Hu, Yan Li, Zhenqiu Ning, Chengyi Wang, Sookja Kim Chung, Yan Huang, Jingbo Sun, Lihua Zhou, Jie Li, Xiao Cheng

**Affiliations:** ^1^ Chinese Medicine Guangdong Laboratory, State Key Laboratory of Traditional Chinese Medicine Syndrome, The Second Affiliated Hospital of Guangzhou University of Chinese Medicine, Guangzhou, Guangdong, China; ^2^ Guangdong Provincial Key Laboratory of Research on Emergency in TCM, Guangzhou, China; ^3^ Department of Neurology, Guangdong Provincial Hospital of Traditional Chinese Medicine, Guangzhou, China; ^4^ Department of Anesthesiology, Guangdong Provincial Hospital of Traditional Chinese Medicine, Guangzhou, China; ^5^ Department of Anesthesiology, Shenzhen People’s Hospital, Shenzhen, China; ^6^ Department of Neurology, Guangxi University of Traditional Chinese Medicine Affiliated International Zhuang Medicine Hospital, Nanning, China; ^7^ Faculty of Medicine, Macau University of Science and Technology, Macao, China; ^8^ Department of Anatomy, Zhong Shan School of Medicine, Sun Yat-sen University, Guangzhou, China

**Keywords:** tetramethylpyrazine, ischemia stroke, astrocytes, endothelin-1, blood-brain barrier, reactive oxygen species

## Abstract

Our study focused on the role of traditional Chinese medicine in stroke therapy, specifically targeting endothelin-1 (ET-1) in astrocytes to alleviate ischemic brain injury. Utilizing oxygen-glucose deprivation/reoxygenation (OGD/R) and middle cerebral artery occlusion (MCAO) models, we mimicked cerebral ischemia in both cell cultures and mice. Tetramethylpyrazine (TMP), a component of the Chinese medicine, was identified as a potential therapeutic agent. It significantly increased cell viability, reduced ET-1 expression, and mitigated OGD-induced astrocyte damage, as shown by *in vitro* experiments with ET-1 siRNA and cell lines overexpressing ET-1. In the MCAO animal model, TMP improved neurological scores, decreased infarct size, and lowered ET-1 levels, thus strengthening the blood-brain barrier and reducing oxidative stress. TMP’s neuroprotective effects were further linked to the upregulation of phosphorylated AKT (p-AKT), indicating that the AKT pathway may function downstream of ET-1. These results highlight TMP’s potential in treating ischemic stroke by modulating the ET-1 and AKT signaling pathways, offering a promising avenue for future stroke therapies.

## Highlights

Tetramethylpyrazine regulates ET-1/Akt pathway in astrocytes.
Tetramethylpyrazine inhibits ROS and oxidative stress of cerebral ischemic injury.
Tetramethylpyrazine attenuates the blood-brain barrier damage.

## 1 Introduction

Stroke ranks as the second leading cause of death globally and a significant cause of disability, exhibiting an increasing incidence in developing countries (Llombart et al., 2013). Ischaemic stroke, resulting from arterial occlusion, accounts for the majority of stroke cases (Tsao et al., 2023). Currently, two treatment strategies for ischemic stroke are available in clinical: pharmacological (tPA intravenous thrombolysis) and surgical (endovascular thrombectomy), both capable of achieving rapid reperfusion and minimizing disability (Campbell et al., 2019). However, the utilization rate of these treatments remains low due to the narrow therapeutic window, bleeding complications, and the potential for ischemia-reperfusion (I/R) injury caused by blood flow recanalization (Vahedi and Bousser, 2002). Therefore, it is crucial to seek effective neuroprotective drugs and understand their specific mechanisms to enhance clinical treatment for ischemic stroke.

Astrocytes are the most abundant cells in the brain and perform adiverse functions (Xie and Yang, 2015). They participate in the maintenance of the blood-brain barrier (BBB) by interacting with pericytes and brain microvascular endothelial cells (BMECs) through their endfeet (Xu et al., 2019). Following stroke, astrocytes are known as reactive astrocytes because they are stimulated by various damage-associated molecular patterns and cytokines, resulting in significant changes in their reactivity, gene expression, and functional characteristics (Li et al., 2022). After ischemic stroke, astrocytes are involved in BBB injury and repair by secreting multiple factors (Gimsa et al., 2013).

Endothelin-1 (ET-1), a 21-amino acid peptide possessing potent vasoconstrictive properties (Reid et al., 1995), is primarily synthesized by BMECs and a diverse array of other cells, including neurons and astrocytes (D'Orléans-Juste et al., 2019). Under physiological conditions, as a component of the BBB, astrocytes hardly express ET-1 and its receptors. However, upon pathological insults such as cerebral ischemia, brain injury, and encephalitis, astrocytes can express all components of the endothelin system, including ET-1, ET_A_, and ET_B_ (Hostenbach et al., 2016). In patients with cerebral ischemia, the levels of ET-1 in cerebrospinal fluid and plasma are significantly elevated, and this increase correlates directly with the severity of neurological deficits, suggesting that ET-1 plays a crucial role in the pathological process of cerebral ischemic injury (Volpe and Cosentino, 2000). Our previous research has revealed that excessive expression of ET-1 in astrocytes can exacerbate neurological deficits and disrupt the blood-brain barrier (Lo et al., 2005). Counteracting the detrimental effects of astrocytic ET-1 may represent a promising therapeutic approach for reducing secondary brain damage in various neurological diseases (Hostenbach et al., 2016). Therefore, we further explore the possibility of screening effective drugs for the treatment of ischemic stroke from traditional Chinese medicine, targeting the expression of ET-1 in astrocytes and improving the prognosis of cerebral ischemia through both *in vivo* and *in vitro* stroke models. In our preliminary experiments, we screened out Tetramethylpyrazine (TMP) among three drugs (the other two being Scutellarin and Tanshinone IIA) using an Oxygen/Glucose Deprivation (OGD) cell model *in vitro*.

*Ligusticum wallichii* Franchat (Chuan Xiong) is a commonly employed ingredient in various traditional Chinese medicinal formulations, exhibiting efficacy in managing diverse central nervous system disorders, thereby suggesting potential neuroprotective properties. TMP, the primary active constituent of *L. wallichii* Franchat (Chuan Xiong), is widely used in the treatment of ischemic brain diseases. Research has demonstrated that TMP can reduce blood-brain barrier (BBB) permeability in cerebral ischemia-reperfusion injury (Tan et al., 2015). However, the question of whether TMP modulates the astrocyte-secreted factor ET-1 to cross the BBB in ischemic stroke remains to be urgently validated. In this study, we aimed to investigate whether TMP protects the BBB against cerebral ischemia injury by downregulating ET-1 secreted by astrocytes, and we further explored the underlying mechanisms.

## 2 Materials and methods

### 2.1 Oxygen/glucose deprivation (OGD) condition

Astrocytes were cultured in Dulbecco’s Modified Eagle Medium (DMEM, Gibco, United States) and 10% fetal bovine serum (FBS, Gibco, Australia), and incubated the cells in humidified atmosphere at 37°C with 5% CO_2_. Astrocytes were seeded in 96-well plates at a density of 5 × 10^3^/mL or six-well at a density of 3 × 10^5^/mL. When the cells became confluent, glucose-free DMEM (Gibco, United States) was used to replace the culture medium, and the cells were put into an anaerobic incubator (94% N_2_, 5% CO_2_, 1% O_2_) for 2, 4, 6, 8 h.

### 2.2 Transfection of siRNA into astrocytes

ET-1 siRNA plasmid, negative control plasmid (NC) and transfection kit were provided by Ribobio Corporation (Guangzhou, China). Astrocytes (ATCC, CRL-2005) were cultured in antibiotic-free medium and incubated with ET-1 siRNA or NC for 24 h, then OGD 6 h was performed with glucose-free medium DMEM.

### 2.3 Culture of astrocyte-like ET-1-overexpressing (C6-ET-1) and mock-transfected clone (C6-Mock) cell lines

The cells were developed following a previously described method (Hung et al., 2015). Specifically, the C6-ET1 cell line was generated by cotransfecting a plasmid that drives the overexpression of the active ET-1 peptide (pGET-1) with a plasmid containing a puromycin resistance gene (pPUR) into a D1 TNC1 astrocytic cell line (ATCC, CRL-2005). The puromycin-resistant clones were screened using PCR and selected based on their resistance to puromycin to ensure stable integration of pGET-1. The stable clone was then characterized and selected based on the level of ET-1 secretion, which was determined using an enzyme-linked immunosorbent assay (ELISA) from Amersham (Piscataway, NJ, United States). A mock-transfected clone (C6-Mock) was used as a control, where pMock (i.e., pGET-1 without ET-1 cDNA and SV40 polyA signal) and pPUR were cotransfected.

### 2.4 Cell counting kit-8 (CCK8) assay

Cell viability was determined using the CCK8 assay (Shanghai Yeasen Technology Co., Ltd., Cat# 40203ES88). All of the procedures followed the manufacturer’s instructions. 90 μL DMEM and 10 μL CCK8 solution were added to each hole of the 96-well plate. After 2 h of incubation at 37°C with 5% CO_2_, The absorbance of the cells was measured at 450 nm by a full-wavelength enzymograph and the cell viability was calculated.

### 2.5 ELISA detection of ET-1

The adherent cells were gently cleaned with cold PBS, then the protein was extracted with a cell scraper, and the cells were collected after 1,000 × g centrifugation for 5 min (min). Centrifuge 1,000 rpm for 5 min, discard the supernatant, add 80 μL PBS, freeze and thaw for five times; The supernatant was extracted after centrifugation at 12,000 rpm at 4°C for 10 min. The protein concentration was determined by BCA method and homogenized. The concentration of ET-1 was determined by ELISA kit (Lot: E-EL-R1458c., Wuhan Elabscience Biotechnology Co., Ltd.).

### 2.6 TMP treatment

*In vitro* study, TMP (Cat: S3956, Purity: 99.96%, Selleck Chemicals Co., United States) with a concentration gradient of 0.01, 0.1, 1, 10, and 100 μM was prepared in DMEM, then 100 μL was added to 96-well plate and cultured in a 5% CO_2_ incubator at 37°C. After 24 h, the toxicity of the drug was evaluated by testing cell viability. TMP with concentration gradients of 0.005, 0.01, 0.05, 0.1, and 0.5 μM were configured in sugar-free medium, and 96-well plate cells were treated with 100 μL during OGD to detect cell viability.

### 2.7 Animals and treatment

In this study, we used wild type (WT) mice (n = 60) and GET-1 transgenic mice (n = 60). Male mice were aged 8–12 weeks, and their weights were kept between 20 and 25 g. WT mice were obtained from the Guangdong Medical Experimental Animal Center (SYXK (Yue) 2018-0094, specific-pathogen-free grade) and raised in the Guangdong Traditional Chinese Medicine Hospital’s Experimental Animal Center. We obtained GET-1 mice from The University of Hong Kong. The Guangzhou University of Chinese Medicine Experimental Animal Center took responsibility for rearing all the mice. They were housed with a fixed temperature and humidity, a 12-h day and night cycle, and *ad lib* access to water and food. Each cage held up to five mice. The Guangdong Provincial Hospital of Traditional Chinese Medicine’s Experimental Animal Ethics Committee approved the animal experiments. Mice in the different categories were labeled and then grouped via the random number method. The WT mice and GET-1 transgenic mice were randomized to the sham (n = 16) and MCAO groups (n = 22), MCAO + TMP groups (n = 22) respectively. In TMP administration group, 12.5 mg/kg and 25 mg/kg TMP dissolved in saline was administered intraperitoneally 20 min before surgery and 12 h after surgery.

### 2.8 MCAO model

We developed the MCAO model as described previously (Cheng et al., 2019). In brief, the mice were anesthetized via inhalation of 3% isoflurane (RIWARD, Shenzhen, China) in 70% N_2_O/30% O_2_ for induction and 1% isoflurane in 70% N_2_O/30% O_2_ for maintenance. Then, the right common carotid artery, external carotid artery, and internal carotid artery were separated. Standardized silicone-coated monofilaments (Guangzhou Jialing Technology Co., Ltd., Cat# 1800AAA) were inserted into the external carotid artery on the right side and then pushed upward into the right internal carotid artery until a sense of resistance to stop was felt. After 1 h, the plug was removed to initiate 1 day of reperfusion. During and following the surgical procedure, utilize a color Doppler blood flow monitor (PeriCam PSI System, United States) to track local cerebral blood flow and validate the successful establishment of the middle cerebral artery occlusion model (Supplementary Figure S1).

### 2.9 Neurological scores

Twenty-four hours after ischemia and reperfusion, Zea-Longa method (Bederson et al., 1986) was used to evaluate the neurological function of the mice, which was divided into 0–4 scale. 0: no nerve injury; 1: adduction flexion of the contralateral forelimb during tail lifting; 2: rotate to the opposite side during crawling; 3: when standing or crawling, falling to the opposite side; 4: no autonomic activity with disturbance of consciousness. The neurological function score was independently completed by two researchers who were blinded to grouping information.

### 2.10 2,3,5-triphenyltetrazolium chloride (TTC) staining

After neurological scoring, the brains of anesthetized mice were taken and divided into five sections along the average thickness of the coronal section. Put into a small black box containing 2% TTC (cat: T8877, Sigma, United States) solution and incubate at 37°C for 15 min. The stained tissue was photographed in a fixed order of segmentation. ImageJ software was used to calculate the cerebral infarction area, and the cerebral infarction area was the ratio of the pale area of the infarction to the total brain area.

### 2.11 BBB permeability evaluation

To evaluate BBB disruption after MCAO, Evans blue leakage into the brain parenchyma was used. Briefly, after anesthesia, the mice’s tail vein was exposed to alcohol, and 0.1 mL of 2%EB solution was injected along it. After 1 h cycle, the heart was perfused with normal saline until clear fluid flowed out of the right auricle to wash the remaining EB from the blood vessels. The cerebral cortex was taken and weighed, 1.5 mL formamide was added and homogenized, and incubated at 55°C for 24 h. The supernatant was collected after centrifugation, and the absorbance was measured at 620 nm to determine the content.

### 2.12 Western blot

A manual grinder was used to homogenize the frozen brain samples immersed in RIPA buffer with protease inhibitors (Cat# 5892791001, Roche) and phosphatase inhibitors (Cat# 04906837001, Roche) inside. Collecting the supernatants and then using the BCA method to measure the protein concentrations. Then the loading buffer was mixed, and heated at 100°C for 10 min. After 8 µL protein samples were taken from each group, PVDF membrane was used to transfer the protein after separation using 12% SDS-PAGE, and then the 5% Bovine Serum Albumin was dissolved in TBST buffer and blocked for 1.5 h. The diluted primary antibody was used to incubate the membrane for 4°C overnight, including ET-1(1:1,000, Cat# ab2786, Abcam), ZO-1(1:1,000, Cat# ab96587, Abcam), occludin (1:1,000, Cat# ab216327, Abcam)and β-Actin(1:2000, Cat# 8457S, Cell Signaling Technology), GAPDH (1:1,000, Cat# T0004, Affinity Biosciences). TBST three times, 10 min each time was used to wash the membrane. After incubation with the corresponding secondary antibody diluted (1:2,000, Cat# 5174S, Cell Signaling Technology) in TBST for 1 h at ambient temperature, TBST was used to wash the membrane and finally, the enhanced chemiluminescence kit (Millipore) was applied to run the Western blot. After obtaining the image results, the gray density value was measured with Image Lab software.

### 2.13 Quantitative real-time polymerase chain reaction (qRT-PCR) detection

RNA was extracted with EZbiotech. The RNA concentration of each group was detected. Following the instructions contained in the Prime ScriptTM RT Reagent Kit (Cat: RR037A, Dalian TaKaRa Biological Technology Co., Ltd.) kit manual, reverse transcription was performed on a PCR instrument to synthesize cDNA from total RNA. Following the kit manual of the SYBR PremixEx Taq TM II (Tli RNaseH Plus) kit (Cat: RR820A, Dalian TaKaRa Biological Technology Co., Ltd.), qRT-PCR was performed to using the reverse transcription product as a template to detect and quantify ET-1, ZO-1 mRNA levels. The primer sequences used for qRT-PCR are listed in Table 1 (Shanghai British Weijieji Trading Co., Ltd.). The reaction conditions were as follow: predenaturation at 95°C for 10 min, followed by 40 cycles of denaturation at 95°C for 15 s and annealing at 65°C for 60 s and extension at 72°C for 60 s, 40 cycles. The mRNA expression level was calculated by the 2^−ΔΔCT^ method. The value for mRNA on the contralateral side 230 served as normal control and was set at 100%.

**TABLE 1:** Primer sequences.

Name	Primer sequences (5′-3′)
ET-1 (Mouse)	Forward primer: TTC​TTG​CCG​GTT​GGG​AAT​GA
	Reverse primer: TTT​CTA​CAG​AAA​CCC​CGC​CC
ZO-1 (Mouse)	Forward primer: AGACGCCCGAGGGTGTAG
	Reverse primer: TGG​GAC​AAA​AGT​CCG​GGA​AG
GAPDH (Mouse)	Forward primer: CCC​TTA​AGA​GGG​ATG​CTG​CC
	Reverse primer: TAC​GGC​CAA​ATC​CGT​TCA​CA
ET-1 (RAT)	Forward primer: GAC​AAA​GAA​CTC​CGA​GCC​CA
	Reverse primer: AGC​TTG​GGA​CAG​GGT​TTT​CC
ZO-1 (RAT)	Forward primer: GTC​TCG​GAA​AAG​TGC​CAG​GA
	Reverse primer: CAG​GGC​ACC​ATA​CCA​ACC​AT
GAPDH (RAT)	Forward primer: GCA​TCT​TCT​TGT​GCA​GTG​CC
	Reverse primer: TAC​GGC​CAA​ATC​CGT​TCA​CA

### 2.14 ROS detection

The level of reactive oxygen species (ROS) was determined using DCFH-DA protocol assay. In brief, after cell treatment, cells were washed with PBS and incubated with 10 μM of DCFH-DA dissolved in the medium at 37°C for 30 min in the dark and then washed thrice with PBS. Cellular fluorescence was measured using a microplate reader at an excitation wavelength of 488 nm.

### 2.15 Superoxide dismutase (SOD), glutathione (GSH-PX) and malondialdehyde (MDA) detection

SOD, GSH and MDA detection kits were used to detect the levels of SOD, GSH-PX and MDA in cells, respectively. In a six-well plate, the cells/Wells were planted and incubated overnight at about 1 × 10^6^, then treated with cresol for 24 h, then washed twice with cold PBS, and the supernatant was collected by centrifugation for 4 min, then followed the kit instructions, and finally tested on an enzyme label.

### 2.16 Statistical analysis

SPSS 25.0 software was used for statistical analysis of the experimental data. All data were tested for normality using the Shapiro-Wilk test, and data that fit the normal distribution were expressed using the mean ± standard deviation (SD). Levene test was used to test the homogeneity of variance. One-way analysis of variance was used to analyze the data among multiple groups conforming to the homogeneity of variance. LSD test was used to compare the data after pair comparison. Univariate analysis of variance was used for those who did not conform to homogeneity of variance, and the Dunnett T3 test with no assumed homogeneity of variance was used for pound-after comparison. The data between the two groups conforming to the homogeneity of variance were statistically analyzed using the two-independent sample t-test. Two independent sample t ‘test was used to analyze the data between the two groups that did not conform to the homogeneity of variance. P < 0.05 indicated that the difference was statistically significant.

## 3 Results

### 3.1 TMP protects astrocytes against OGD injury and downregulates OGD-induced ET-1 overexpression *in vitro*

**FIGURE 1:**
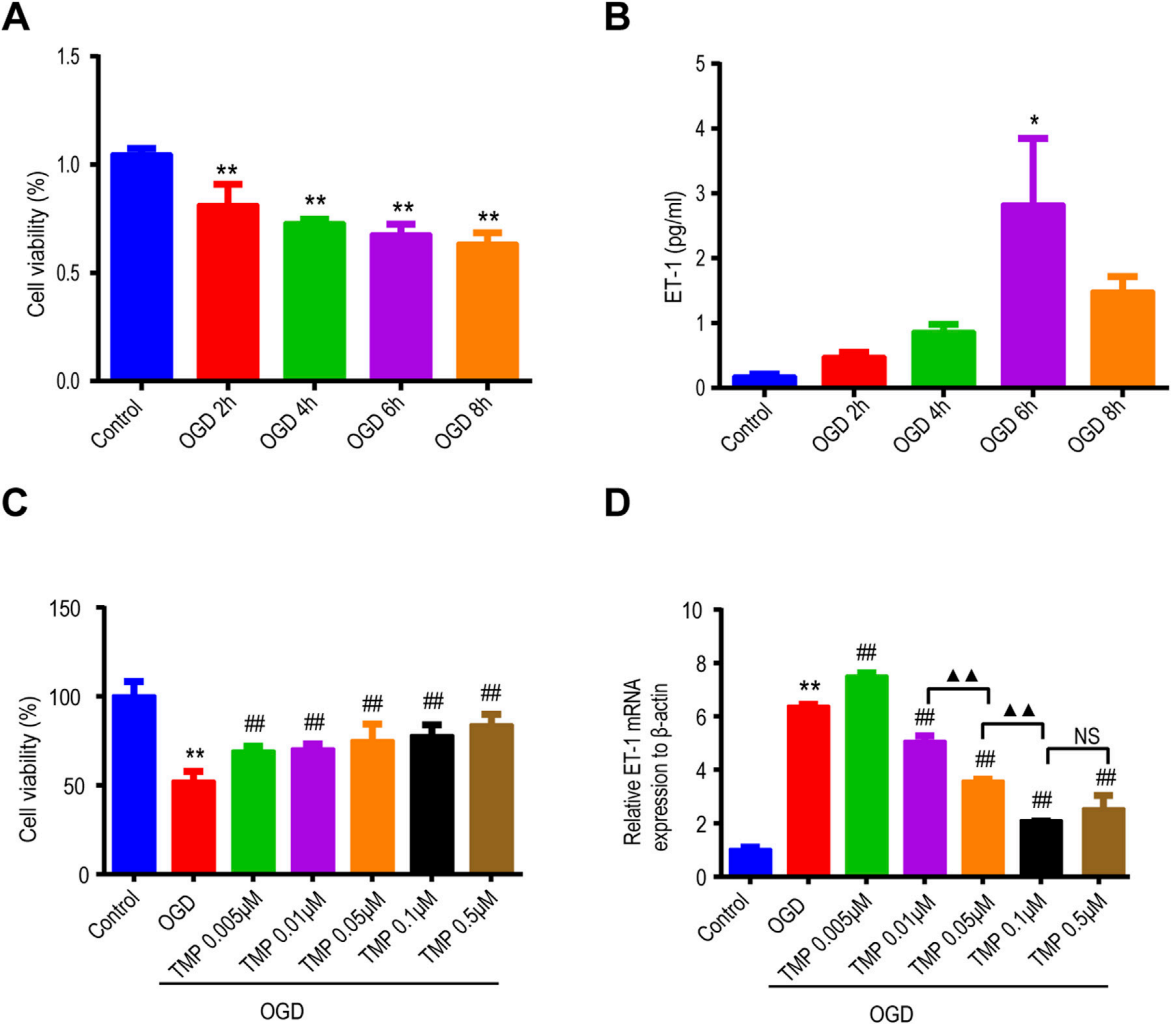
TMP protects astrocytes against OGD injury and reduces the overexpression of ET-1 induced by OGD *in vitro*. **(A, B)** The viability of astrocytes (n = 5) and the expression of ET-1 protein (n = 3) vary with the duration of OGD exposure. **(C, D)** After different concentrations of TMP treatment, the viability of OGD-injured astrocytes (n = 12) and the expression of ET-1 mRNA (n = 3) exhibit changes. *P < 0.05, **P < 0.01 vs. Control; ^##^P < 0.01 vs. OGD; ▲▲P < 0.01, NS represents P > 0.05. TMP, tetramethylpyrazine; OGD, Oxygen/glucose deprivation; ET-1, endothelin-1.

To observe the viability of astrocytes and ET-1 expression after OGD injury, cell viability was determined by CCK8 assay and ET-1 expression was detected by ELISA in cultured astrocytes. The results showed that with the increase of OGD time, cell viability decreased (Figure 1A). ET-1 expression increased with the extension of OGD time and that was significantly increased at 6 h after OGD compared with the normal control group (Figure 1B), therefore, we chose the OGD 6 h injury model to further observe the effect of TMP on OGD injury and ET-1 expression. TMP (0.01–100 μM) concentrations had no toxic effects on normal astrocytes (Supplementary Figure S2). Different concentrations of TMP (0.005, 0.01, 0.05, 0.1 and 0.5 μM) were administered to OGD injured astrocytes for 6 h, the results revealed a significant decrease in the cell viability of astrocytes in the OGD group compared to the normal control group. Notably, TMP increased the viability of the OGD-injured astrocytes in a dose-dependent manner (Figure 1C). qPCR analysis showed that ET-1 mRNA was significantly increased at 6 h after OGD compared with the normal control group, while TMP downregulated ET-1 mRNA levels induced by OGD injury, and the most significant downregulation was observed at a concentration of 0.1 μM (Figure 1D). The above results indicate that TMP can protect astrocytes damaged by OGD and downregulate ET-1 levels in astrocytes induced by OGD.

### 3.2 TMP alleviated OGD-induced astrocyte injury by suppressing ET-1 *in vitro*

**FIGURE 2:**
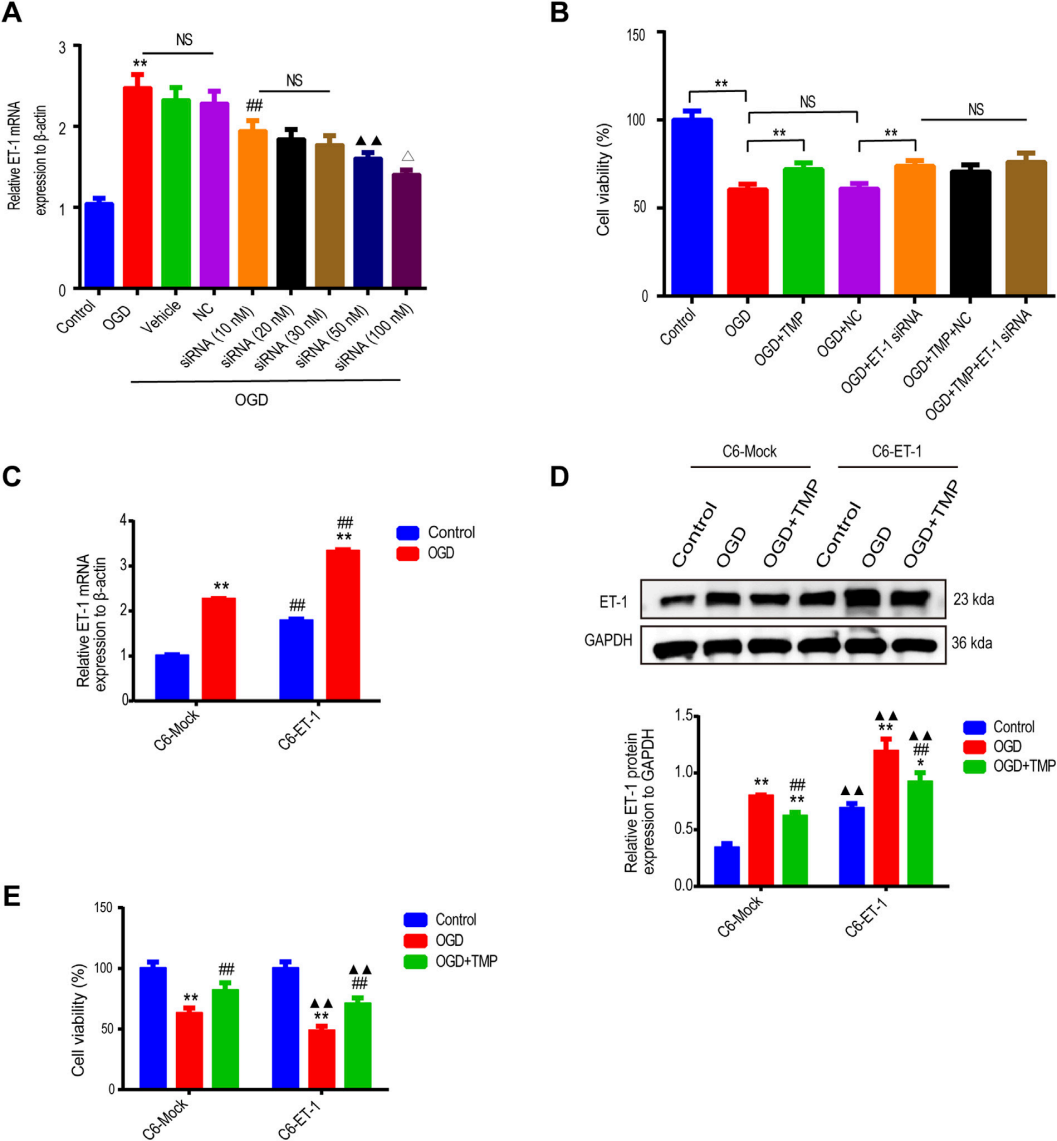
The protective effect of TMP against OGD injury is intricately linked to astrocytic ET-1 **(A)** The ET-1 mRNA expression in OGD-injured astrocytes following the application of varying concentrations of ET-1 siRNA (n = 6). **P < 0.01 vs. Control, ^##^P < 0.01 vs. NC, ▲▲P < 0.01 vs. siRNA (10 nM), ^∆^P < 0.05 vs. siRNA (50 nM), NS represents P > 0.05. **(B)** The cell viability of astrocytes in different treatment groups (n = 6). **P < 0.01, NS represents P > 0.05. **(C)**, The ET-1 mRNA expression of OGD injured C6-Mock and C6-ET-1 (n = 6). Comparison within the same cell lines: **P < 0.01 vs. OGD; Comparison between different cell lines: ^##^P < 0.01 vs. C6-Mock **(D, E)**, The ET-1 expression (n = 3) and cell viability (n = 12) in different treatment groups of C6-Mock and C6-ET-1. Comparison within the same cell lines: *P < 0.05, **P < 0.01 vs. Control, ^##^P < 0.01 vs. OGD; Comparison between different cell lines: ▲▲P < 0.01 vs. C6-Mock. TMP, tetramethylpyrazine; OGD, Oxygen/glucose deprivation; ET-1, endothelin-1; C6-Mock, Mock-transfected clone; NC, negative control plasmid; ns, no significant; C6-ET-1, astrocyte-like ET-1-overexpressing.

To confirm whether the protective effect of TMP against OGD injury is directly associated with astrocytic ET-1, we regulated the expression of ET-1 in astrocytes through the use of ET-1 siRNA *in vitro* and then observed the effect of TMP on OGD injury. Initially, we assessed if ET-1 siRNA could effectively downregulate ET-1 mRNA expression in OGD-injured astrocytes We first investigated whether ET-1 siRNA could effective downregulate ET-1 mRNA expression in OGD injured astrocytes, LipofectamineTM2000 buffer (vehicle control), a negative control plasmid (NC), or various concentrations (10, 20, 30, 50, 100 nM) of ET-1 siRNA were administered to OGD-injured astrocytes, and qPCR was employed to analyze ET-1 mRNA levels in each group’s astrocytes. The results indicated that ET-1 mRNA levels in cells treated with 100 nM ET-1 siRNA were significantly reduced compared to the vehicle control group or NC control group after OGD, while no significant difference was observed among OGD group, the vehicle control group post-OGD and NC groups post-OGD (Figure 2A). This suggests that astrocytes transfected with 100 nM ET-1 siRNA can effectively reduce ET-1 mRNA expression following OGD, thus we selected 100 nM ET-1 siRNA for further experimentation. Furthermore, we investigated the protective effect of TMP on OGD-induced astrocyte injury following TMP and/or ET-1 siRNA treatment. The results revealed that the cell viability of astrocytes decreased in the OGD group compared to the normal control group, while the viability of astrocytes in the TMP + OGD, ET-1 siRNA + OGD, and TMP + OGD + ET-1 siRNA groups were enhanced compared to the OGD group. However, no significant difference was observed among TMP + OGD, ET-1 siRNA + OGD and TMP + OGD + ET-1 siRNA groups (Figure 2B). The above results indicate that TMP can exert the efficacy of ET-1 inhibitor in OGD-injured astrocytes. When TMP and ET-1 siRNA are administered together, TMP does not exert a stronger protective effect on OGD injury due to the inhibition of ET-1 target, so we concluded that the protective effect of TMP against OGD injury is directly attributed to targeting astrocyte ET-1 *in vitro*.

We subsequently cultured astrocytic cells, both with and without ET-1 overexpression (C6-ET-1 cells and C6-Mock cells), under both normal and oxygen-glucose deprivation (OGD) conditions. Under normal conditions, the baseline levels of ET-1 mRNA and protein in the C6-ET-1 cells were significantly elevated compared to those in the C6-Mock cells. Following 6 h of OGD treatment, a pronounced induction of ET-1 mRNA and protein was observed in both cell lines, with a more substantial increase noted in the C6-ET-1 cells than in the C6-Mock cells (Figure 2C). Notably, the administration of TMP significantly reduced ET-1 mRNA and protein expression and concomitantly enhanced cell viability in both cell lines under OGD conditions (Figures 2D, E). These findings collectively suggest that the protective effect of TMP against OGD injury is directly mediated through targeting astrocytic ET-1 *in vitro*.

### 3.3 TMP mitigates the more severe brain damage caused by astrocytic ET-1 overexpression in MCAO-induced cerebral ischemia injury *in vivo*

As we know, mice deficient in ET-1, ETA, or ETB receptors exhibit lethal phenotypes (Baynash et al., 1994; Clouthier et al., 1998). In our previous study, we demonstrated that mice overexpressing astrocytic ET-1 (GET-1) exhibited more severe neurological deficits coupled with BBB integrity impairment, which contributed significantly to more severe ischemic brain injury (Lo et al., 2005). Therefore, in this study, we further investigated whether TMP protects the brain from damage caused by ET-1 overexpression in astrocytes by reducing ET-1 expression *in vivo*. Administration of TMP at 12.5 mg/kg and 25 mg/kg effectively reduced ET-1 protein levels in brain tissue after MCAO, with the downregulation effect of 25 mg/kg being more significant. Hence, 25 mg/kg was chosen as the intervention concentration for TMP for further study (Supplementary Figure S3).

**FIGURE 3:**
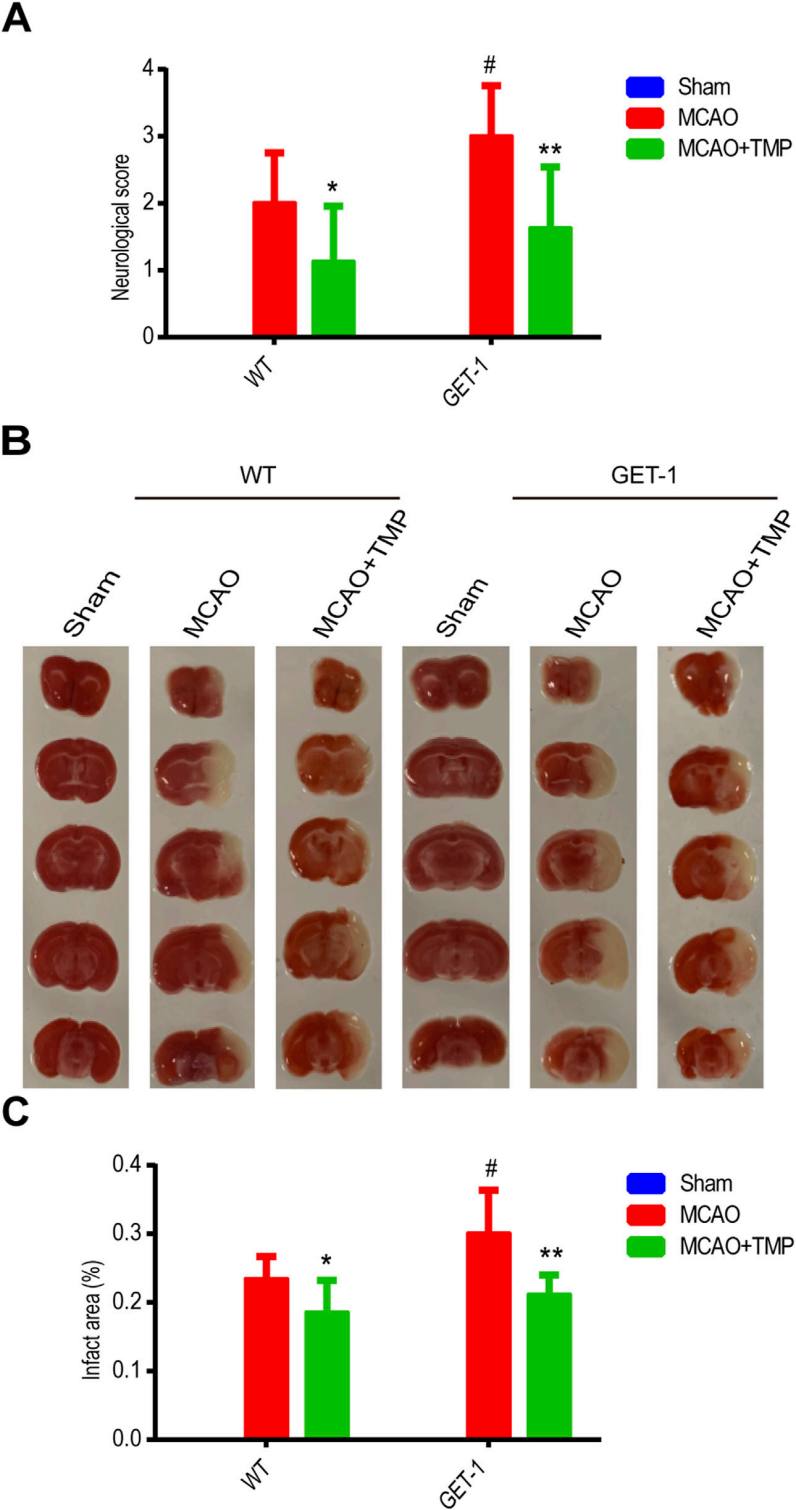
TMP mitigates the more severe neurological deficits and infarct area caused by astrocytic ET-1 overexpression in MCAO-induced cerebral ischemia injury. Neural function score **(A)** and infarct areas of different treatment groups **(B, C)** (n = 8). Comparison within the same genotype mice, *P < 0.05, **P < 0.01 vs. MCAO; Comparison between WT and GET-1 mice, ^#^P < 0.05 vs. WT. TMP, tetramethylpyrazine; MCAO, middle cerebral artery occlusion; ET-1, endothelin-1; WT, Wild-type mice; GET-1, astrocytic endothelial-1 overexpression mice.

In Figure 3, we observed that there was no neurological function deficit or infarct area in the sham group, regardless of whether it comprised WT or GET-1 mice. Conspicuously, the neural function score and infarct area in the MCAO group were significantly higher compared to the Sham group, both in WT and GET-1 mice. Notably, the values for GET-1 mice in the MCAO group exceeded those of WT mice, aligning with our previous study. Intriguingly, the neurological function deficit and infarct area in the TMP group were significantly reduced compared to the MCAO group, both in WT and GET-1 mice (Figures 3A–C). The results suggest that TMP ameliorates neurological deficits and reduces the cerebral infarction area following MCAO. Moreover, in scenarios where MCAO-induced cerebral ischemia is exacerbated by the overexpression of ET-1 in astrocytes, TMP exhibits a more profound ability to alleviate severe neurological deficits and substantial infarct areas.

**FIGURE 4:**
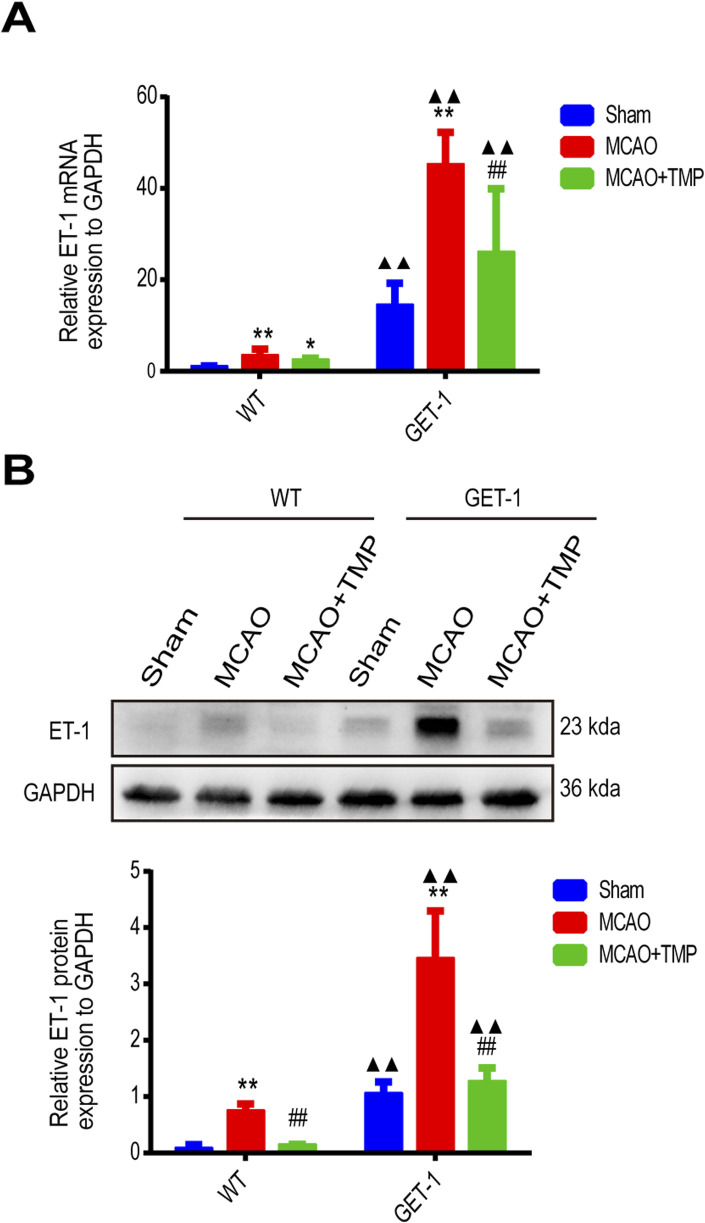
TMP decrease MCAO induced ET-1 mRNA and protein expression. **(A, B)** The ET-1 mRNA expression (n = 6) and ET-1 protein expression (n = 4) of different treatment groups in WT and GET-1 mice. Comparison within the same genotype mice, *P < 0.05, **P < 0.01 vs. Sham; ^##^P < 0.01 vs. MCAO; Comparison between WT and GET-1 mice, ▲▲P < 0.01 vs. WT. TMP, tetramethylpyrazine; MCAO, middle cerebral artery occlusion; ET-1, endothelin-1; WT, Wild-type; GET-1, astrocytic ET-1 overexpression mice.

The qPCR and Western blot analysis of ET-1 mRNA and protein levels in ischemic brain tissue revealed almost no ET-1 expression in the WT-Sham group, whereas a marked increase was observed in the GET-1-Sham group. Notably, the ET-1 mRNA and protein expression levels in the MCAO groups were significantly elevated compared to the Sham group, with the TMP group exhibiting lower levels than the MCAO group, both in WT and GET-1 mice. Additionally, GET-1 mice in the MCAO group displayed higher ET-1 mRNA and protein expression compared to WT mice (Figures 4A, B). This indicates that TMP administration significantly mitigates MCAO-induced ET-1 overexpression in both WT and GET-1 mice.

### 3.4 TMP repairs more severe blood-brain barrier damage caused by ET-1 overexpression in MCAO mice

**FIGURE 5:**
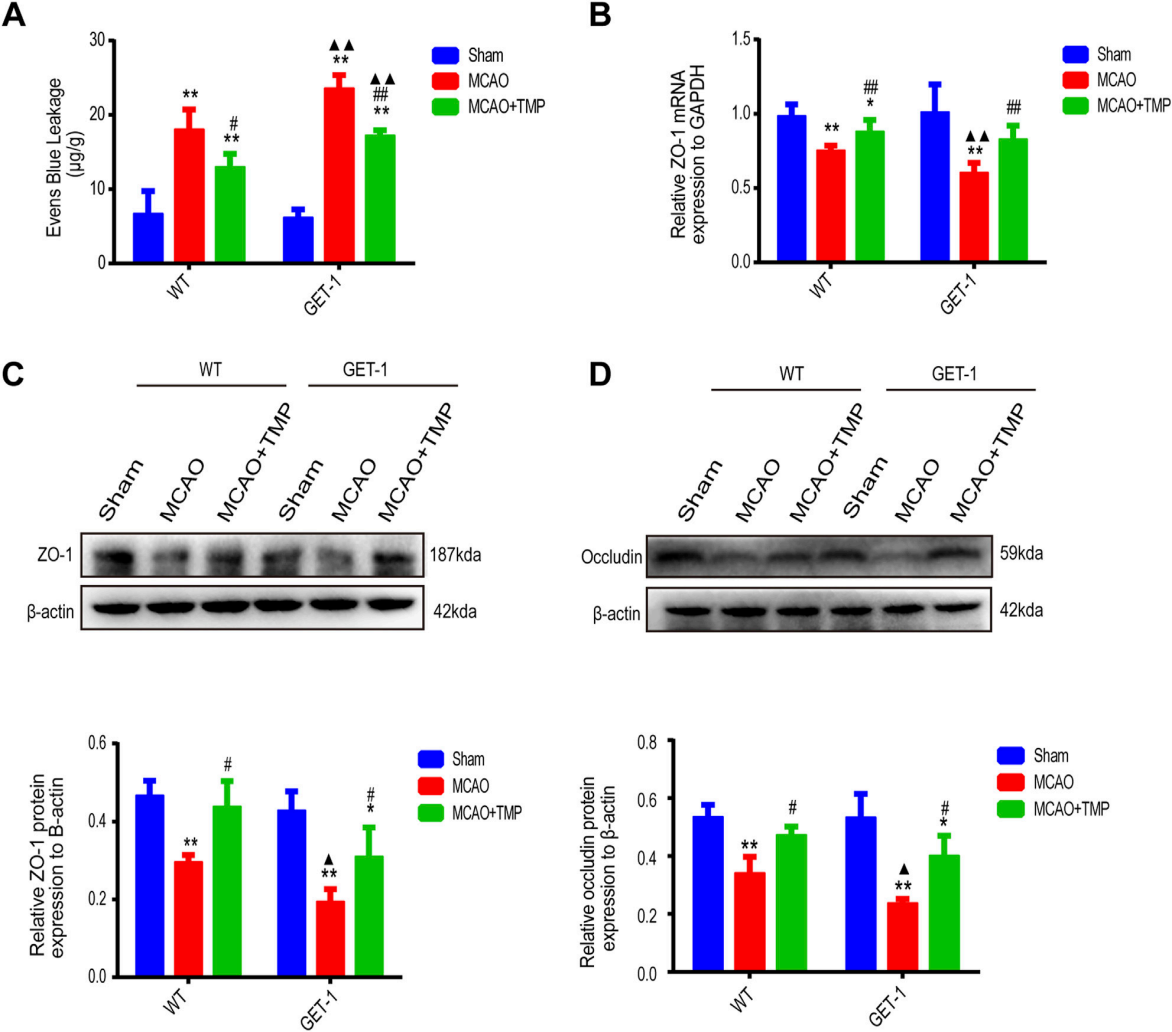
TMP repairs more severe blood-brain barrier damage caused by ET-1 overexpression in MCAO mice. The EB infiltration (**(A)**, n = 5), ZO-1 mRNA expression (**(B)**, n = 6), ZO-1 protein expression (**(C)**, n = 3) and occludin protein expression (**(D)**, n = 3) of different treatment groups in WT and GET-1 mice. Comparison within the same genotype mice, *P < 0.05, **P < 0.01 vs. Sham, #P < 0.05, ^##^P < 0.01 vs. MCAO; Comparison between WT and GET-1 mice, ▲P < 0.05, ▲▲P < 0.01 vs. WT. TMP, tetramethylpyrazine; MCAO, middle cerebral artery occlusion; BBB, blood-brain barrier; EB, evens blue; WT, Wild-type; GET-1, astrocytic ET-1 overexpression.

In both WT and GET-1 mice, quantitative analysis of Evans Blue (EB) infiltration revealed reduced EB exudation in the Sham group compared to significantly higher levels in the MCAO and TMP groups. However, the TMP group exhibited lower EB exudation than the MCAO group. Notably, the EB exudation in GET-1 mice from the MCAO and MCAO + TMP groups was higher than that in WT mice (Figure 5A).

qPCR detection of ZO-1 in ischemic brain tissue of both WT and GET-1 mice showed significantly lower expression in the MCAO and TMP groups compared to the Sham group. Additionally, the TMP group exhibited higher ZO-1 expression than the MCAO group. While GET-1 mice in the MCAO group displayed higher ZO-1 expression than WT mice, no significant difference was observed between the TMP groups (Figure 5B).

Western blot (WB) analysis of ZO-1 and occludin in ischemic lateral brain tissue demonstrated significantly lower expression in the MCAO and TMP groups compared to the Sham group in both WT and GET-1 mice. However, the TMP group exhibited higher expression of both ZO-1 and occludin than the MCAO group. GET-1 mice in the MCAO group displayed higher expression of ZO-1 and occludin compared to WT mice, but no significant difference was observed between the TMP groups (Figures 5C, D). These findings suggest that ET-1 can exacerbate blood-brain barrier (BBB) injury, whereas TMP administration can protect the BBB against cerebral ischemia-reperfusion injury.

### 3.5 TMP demonstrates the ability to suppress ROS and oxidative stress products both *in vivo* and *in vitro* following ischemia stroke injury

**FIGURE 6:**
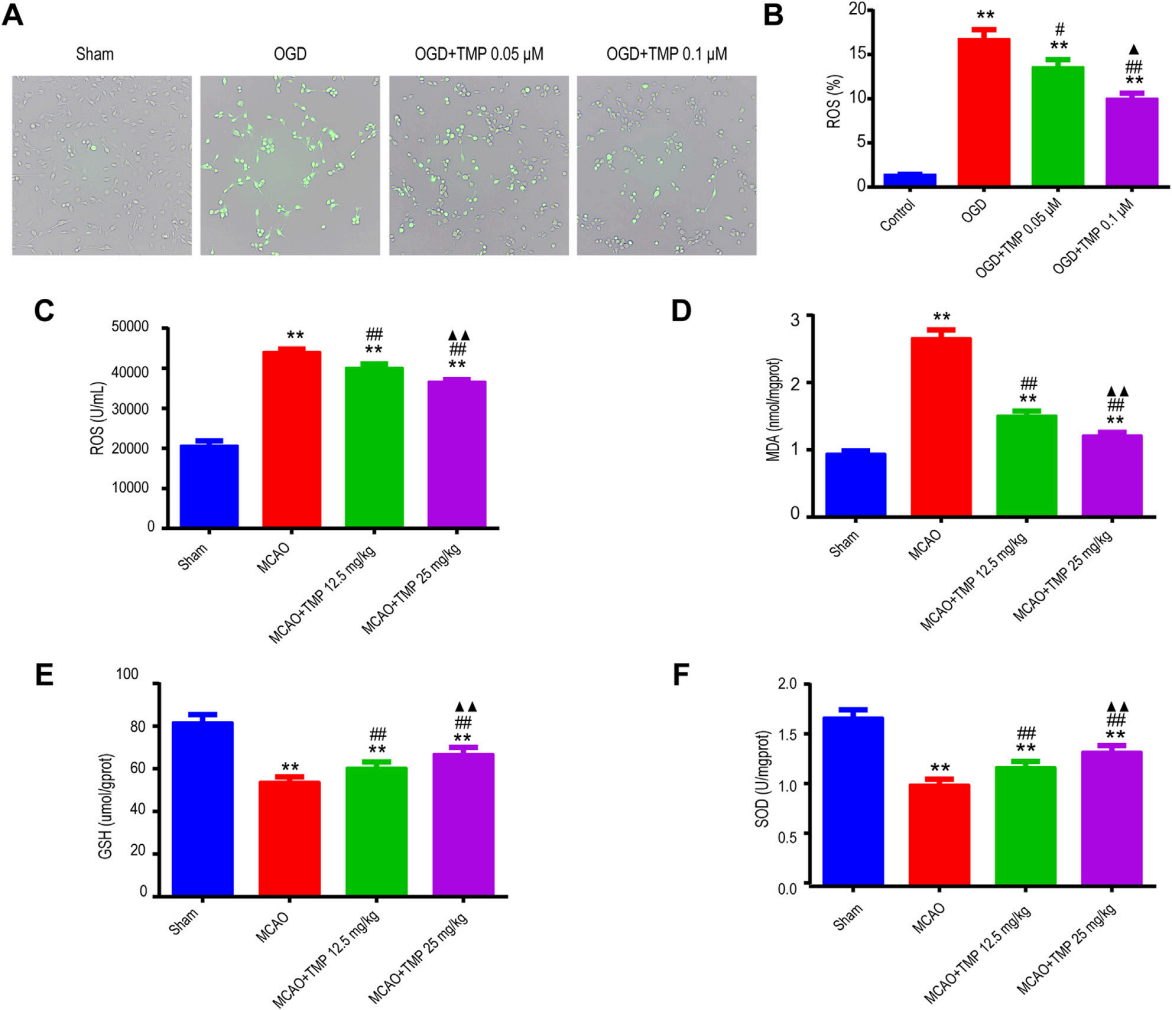
TMP suppressed ROS and oxidative stress products both *in vivo* and *in vitro* following cerebral ischemia injury. **(A, B)** ROS expression of each group of astrocytes (n = 3). **P < 0.01 vs. Control, ^#^P < 0.05, ^##^P < 0.01 vs. OGD, ▲P < 0.05 vs. OGD + TMP 12.5 mg/kg. **(C–F)** ROS, MDA, GSH and SOD expressions of different treatment groups in WT mice (n = 12). **P < 0.01 vs. Sham; ^##^P < 0.01 vs. MCAO; ▲▲P < 0.05 vs. MCAO + TMP 12.5 mg/kg. TMP, tetramethylpyrazine; MCAO, middle cerebral artery occlusion; ROS, reactive oxygen species; MDA, malondialdehyde; GSH, glutathione; SOD, superoxide dismutase; WT, Wild-type; OGD, Oxygen/glucose deprivation.

As is widely known, TMP possesses antioxidant properties. Following ischemia injury, ROS and oxidative stress products were evaluated both *in vivo* and *in vitro*. Fluorescence analysis revealed an increase in ROS expression in astrocytes after OGD injury, whereas TMP administration significantly reduced ROS expression, particularly at a concentration of 0.1 μM *in vitro* (Figures 6A, B). Furthermore, following MCAO injury *in vivo*, the expressions of ROS and MDA were upregulated, while GSH and SOD levels were downregulated compared to the sham group. However, TMP administration reversed these trends, demonstrating a reduction in ROS and MDA expressions and an increase in GSH and SOD levels, with the most significant effect observed at a dose of 25 mg/kg (Figures 6C–F). This suggests that TMP effectively inhibits ROS and oxidative stress products.

### 3.6 TMP protects against ischemia injury by targeting ET-1/Akt pathway in astrocyte

**FIGURE 7:**
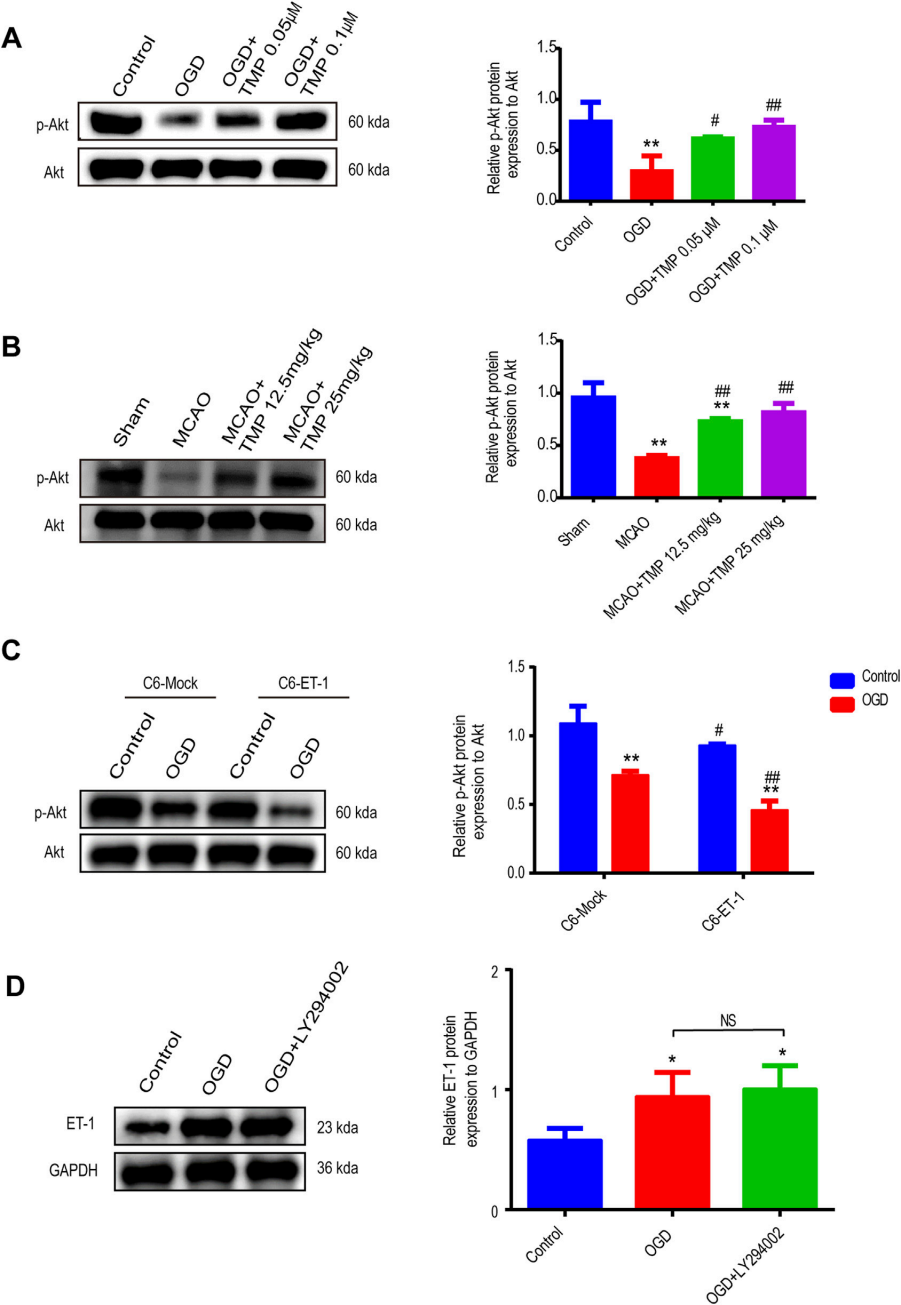
TMP protect against ischemia injury by through ET-1/Akt pathway in astrocytes. **(A)** The p-Akt and ET-1 protein expression of each group in astrocytes (n = 3). **P < 0.01 vs. Control, ^#^P < 0.05, ^##^P < 0.01 vs. OGD. **(B)** The p-Akt protein expression of each group of mice (n = 3). **P < 0.01 vs. Sham, ^##^P < 0.01 vs. MCAO. **(C)** The p-Akt protein expression of different treatment groups in C6-Mock and C6-ET-1(n = 3). Comparison within the same cell lines, **P < 0.01 vs. Control, ^#^P < 0.05, ^##^P < 0.01 vs. OGD. **(D)** The p-Akt protein expression of different treatment groups in C6-Mock (n = 3). *P < 0.05. TMP, tetramethylpyrazine; ET-1, endothelin-1; C6-Mock, Mock-transfected clone; C6-ET-1, astrocyte-like ET-1-overexpressing cells; OGD, Oxygen/glucose deprivation; WT, Wild-type; MCAO, middle cerebral artery occlusion.

To further investigate how TMP modulates the expression of ET-1 in astrocytes and identify its downstream signaling molecules under ischemic conditions, we evaluated Akt protein activation in astrocytes exposed to OGD for 6 h *in vitro* and in an MCAO-induced cerebral ischemia model *in vivo* using Western blot assay (Figures 7A, B). The results indicated no significant difference in Akt expression between the normal control, OGD, 0.05 μM TMP, and 0.1 μM TMP administration groups. However, p-Akt expression was reduced in the OGD group compared to the normal control group, which was significantly elevated following the administration of 0.05 μM and 0.1 μM TMP compared to the OGD model group. Notably, the upregulation of p-Akt was more pronounced in the 0.1 μM TMP administration group (Figure 7A). Similar results were observed in the *in vivo* MCAO model, where there was no significant difference in Akt expression between the sham group, MCAO model group, 12.5 mg/kg TMP, and 25 mg/kg TMP administration groups. However, p-Akt expression was significantly reduced in the MCAO group compared to the sham group, which was markedly increased after the administration of 12.5 and 25 mg/kg TMP compared to the MCAO model group (Figure 7B). Specifically, 25 mg/kg TMP treatment exhibited a more profound effect on Akt phosphorylation activation *in vivo*.

To further clarify the upstream and downstream relationship between ET-1 and Akt signaling, we initially examined Akt phosphorylation activation *in vitro*, comparing an astrocytic ET-1 overexpressing stable cell line (C6-ET-1) with normal cultured astrocytic cells (C6-Mock). Our findings revealed that in the sham group, C6-ET-1 cells expressed lower levels of p-Akt compared to C6-Mock cells. Following OGD injury, p-Akt expression decreased more in C6-ET-1 cells (Figure 7C). Subsequently, we investigated ET-1 expression using the AKT pathway inhibitor LY294002 *in vitro* and observed that the expression of ET-1 in normal C6-Mock cells under OGD conditions remained unaffected following the administration of the AKT inhibitor (Figure 7D). These results suggest that the Akt pathway functions downstream of ET-1 following OGD injury, confirming that TMP can protect against cerebral ischemia-reperfusion injury through the ET-1/AKT pathway in astrocytes.

## 4 Discussion

In our previous studies, transgenic mice that overexpressed astrocytic ET-1 exhibited more profound neurological dysfunction and blood-brain barrier impairment (Lo et al., 2005). This current study marks the first discovery that TMP protects the BBB against cerebral ischemia-reperfusion injury by modulating ET-1/Akt in astrocytes and suppressing ROS and oxidative stress. This significant finding advances our understanding of the pathogenesis of cerebral ischemic injury and paves the way for novel drug targets and therapeutic strategies for treating cerebral ischemic injury.

Astrocytes, a prevalent type of glial cells in the central nervous system, constitute a crucial part of the neurovascular unit. They secrete diverse cytokines to support nerve function, facilitate intercellular transmitter uptake and signaling, and maintain metabolic and homeostatic balance. Following ischemia-reperfusion injury, astrocytes contribute significantly to the uptake of neuroexcitatory toxic ions like glutamate and calcium, while also playing a vital role in regulating the repair of neurological function damage. Astrocytes are the primary cell type in the brain that produces ET-1 (MacCumber et al., 1990). After ischemic brain injury, an increase in ET-like immunoreactivity has been observed in astrocyte-like cells (Kuwaki et al., 1997). A previous study revealed that overexpression of ET-1 in astrocytes exacerbated ischemic brain injury (Lo et al., 2005). Furthermore, it promotes the proliferation of neural progenitor cells and their differentiation into astrocytes via the Jak2/Stat3 pathway (Cheng et al., 2019). Additionally, the PI3K/Akt signaling pathway, as one of the key cascades signaling pathways, is intricately involved in the occurrence and progression of ischemic stroke, intertwining with various mechanisms such as apoptosis, autophagy, oxidative stress, and inflammatory response (Feng Y. et al., 2014). Akt serves as a core effector of the PI3K/Akt signaling pathway. Research has demonstrated that activating Akt phosphorylation can effectively reduce apoptosis induced by ischemia-reperfusion (I/R) (Wang et al., 2009). Additionally, ET-1-mediated inhibition of insulin action has been attributed to its suppression of insulin-stimulated Akt phosphorylation (Horinouchi et al., 2016). The regulation of pre-differentiated human mesenchymal stem cells by ET-1 is closely linked to Akt signaling pathway-mediated mechanisms (Tsai et al., 2015). These findings suggest a close physiological interplay between ET-1 and Akt, predicting a potential signaling role between them in ischemic stroke. In this experiment, GET-1 mice were employed as the research subjects, with WT mice serving as the control. Both groups underwent middle cerebral artery occlusion (MCAO), resulting in a significant increase in ET-1 expression and a concurrent inhibition of Akt phosphorylation. Notably, this deterioration was more severe in GET-1 mice compared to WT mice. Consistent results were also observed in our *in vitro* experimental validation. These findings indicate that during ischemic stroke, the secretion of ET-1 by astrocytes abnormally increases, thereby impeding the physiological function of Akt.

TMP, an amide alkaloid extracted from Chuanxiong, possesses diverse pharmacological effects. These include vasodilating blood vessels, improving tissue microcirculation, inhibiting platelet adhesion and aggregation, regulating lipid metabolism, as well as suppressing the proliferation of smooth muscle cells and fibroblasts. TMP is widely utilized in the treatment of occlusive cerebrovascular diseases (Wang et al., 2013). In the context of stroke therapy, safeguarding the neurovascular unit is paramount (Zagrean et al., 2018). To this end, preserving the integrity of the blood-brain barrier (BBB) is a critical strategy in combating cerebral ischemia/reperfusion injury. TMP aids in neurological function recovery after ischemic stroke by protecting the BBB’s integrity and alleviating cerebral infarction (Xiao et al., 2010; Gong et al., 2019). Furthermore, TMP can counteract astrocyte stress in rats with middle cerebral artery occlusion (MCAO) (Liao et al., 2004). Although TMP has been reported to alleviate vasculopathy during coronary vasoconstriction, our current study reveals that TMP safeguards the BBB’s integrity in the MCAO model and reduces ET-1 secretion resulting from astrocyte stress (Zeng et al., 1998). This suggests a correlation between TMP’s protective mechanism against MCAO and ET-1 secreted by astrocytes. The obstruction of arterial blood supply during the MCAO model’s preparation leads to a significant imbalance in brain tissue metabolism. The imbalance triggered by the MCAO model leads to hypoxia in brain tissue. Upon reperfusion, the restoration of blood flow and oxygenation paradoxically intensifies tissue damage. Neurons, previously operating under anaerobic glycolysis during ischemia and hypoxia, rapidly switch back to aerobic respiration, resulting in the generation of a significant amount of reactive oxygen species (ROS). This, in turn, causes devastating injuries to neurons (Pan et al., 2021). The production of ROS is a contributory factor to vasoconstriction, mediated in part by the effects of endothelin-1 (ET-1) (Chabrashvili et al., 2003). The PI3K/AKT signaling pathway plays a pivotal role in regulating neuronal apoptosis following hypoxic-ischemic injury (Song et al., 2019) and also contributes to improving endothelial function and mitigating atherosclerosis (Xing et al., 2015). This indicates that ROS, ET-1, and AKT are functionally intertwined in the context of MCAO. Our experiment validates the correlation between astrocyte ET-1 and AKT. Specifically, by modulating the expression of ET-1 or AKT, we observed that ET-1 can inversely regulate the phosphorylation of AKT in the MCAO model. This further clarifies the significance of the ET-1/AKT signaling pathway in MCAO. Subsequently, when TMP was administered to treat MCAO, the experimental results revealed that TMP exerted neuroprotective effects by downregulating ET-1 and upregulating AKT after hypoxic-ischemic injury. This elucidates that the neuroprotective pharmacological mechanism of TMP in MCAO involves modulating the ET-1/AKT pathway in astrocytes.

Our findings indicate that TMP protects against cerebral ischemia-reperfusion injury by inhibiting ET-1. Consistent with previous studies, TMP has been shown to reduce pulmonary hypertension by suppressing ET-1 expression (Zhang et al., 2020). Additionally, in a two-kidney-two-clip model of renal vascular hypertension, TMP attenuates basilar artery remodeling, reduces ET-1 and Ang II levels, and elevates NO levels. While TMP can effectively inhibit the activation of the PI3K/Akt pathway, the activation of this pro-survival kinase signaling cascade during reperfusion favors cell survival and triggers anti-apoptotic pathways (Zhang et al., 2020; Yang et al., 2019). Interestingly, some research suggests that TMP may exert a protective role in reducing ischemia-reperfusion-induced apoptosis by activating Akt through phosphorylation (Ding et al., 2019). Our study aligns with previous findings that ET-1 can modulate AKT. Specifically, we demonstrate that TMP inhibits cerebral ischemia-reperfusion injury via the ET-1/AKT pathway. However, it is noteworthy that high doses of TMP can inhibit cell viability and induce apoptosis by generating ROS, leading to the activation of AMP-activated protein kinase (AMPK) (Yi et al., 2013). Furthermore, TMP reduces ET-1 gene expression by inhibiting ROS production induced by angiotensin II (Lee et al., 2005; Wong et al., 2007). In summary, our results provide further insights into the neuroprotective mechanisms of TMP in cerebral ischemia-reperfusion injury, highlighting its potential as a therapeutic agent.

## 5 Conclusion

**FIGURE 8:**
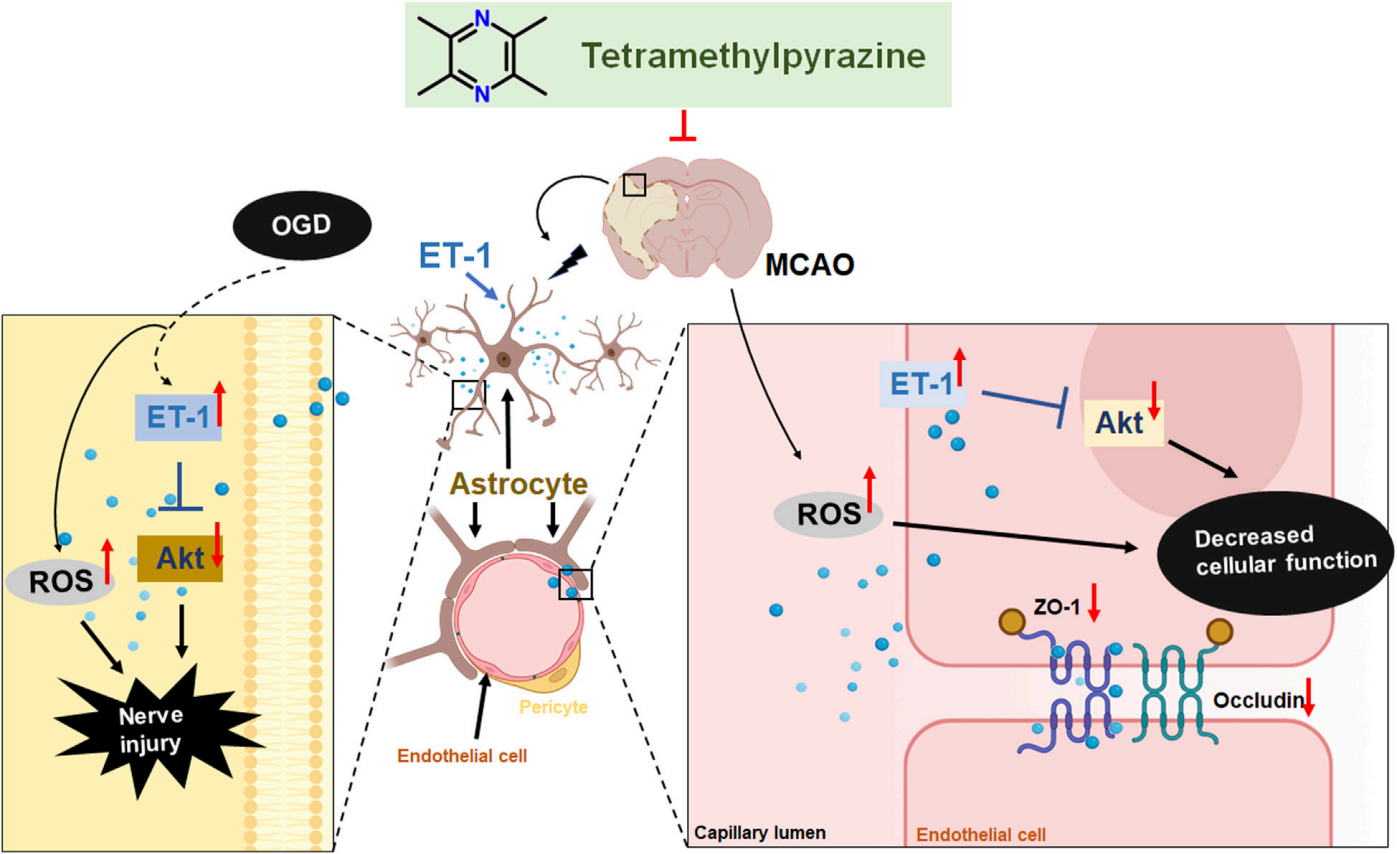
TMP protect MCAO induced BBB damage by targeting endothelin-1/Akt pathway in astrocytes.

These findings offer the first evidence that TMP can safeguard the blood-brain barrier (BBB) against cerebral ischemia-reperfusion injury by modulating ET-1/Akt signaling in astrocytes and suppressing reactive oxygen species (ROS) and oxidative stress. This discovery presents novel drug targets and treatment approaches for cerebral ischemic injury (Figure 8).

## Data Availability

The original contributions presented in the study are included in the article/Supplementary Material, further inquiries can be directed to the corresponding authors.

## Supplementary material

SUPPLEMENTARY FIGURE S1 The cerebral blood flow image changes in WT and GET-1 mice. WT, Wild-type; MCAO, middle cerebral artery occlusion; GET-1, astrocytic ET-1 overexpression.

SUPPLEMENTARY FIGURE S2 The toxicity detection of TMP at different concentrations in astrocytes. TMP, tetramethylpyrazine; ns, no significant.

SUPPLEMENTARY FIGURE S3 The ET-1 protein expression of different treatment groups in WT mice (n = 3). *P < 0.05, **P < 0.01 vs. Sham; ^#^P < 0.05, ^##^P < 0.01 vs. WT MCAO; ▲▲ P < 0.01 vs. MCAO + TMP 12.5 mg/kg. WT, Wild-type; MCAO, middle cerebral artery occlusion; TMP, tetramethylpyrazine; ET-1, endothelin-1.

## Abbreviations

Ang, angiotenisn; AS, Astrocytes; BBB, blood-brain barrier; BMECs, brain microvascular endothelial cells; C6-ET-1, astrocyte-like ET-1-overexpressing; C6-Mock, Mock-transfected clone; CCK8, Cell counting kit-8; EB, evans blue; ELISA, enzyme-linked immunosorbent assay; ET-1, endothelin-1; GET-1, astrocytic ET-1 overexpression; GSH, glutathione; h, hours; KO, knockdown; MCAO, middle cerebral artery occlusion; MDA, malondialdehyde; min, minutes; NC, negative control; ns, no significant; OGD, Oxygen/glucose deprivation; ROS, reactive oxygen species; rtPA, recombinant tissue plasminogen activator; SD, standard deviation; SOD, superoxide dismutase; TMP, tetramethylpyrazine; WT, Wild-type.
