# Supplementary figures and images for: Tetramethylpyrazine attenuates the blood-brain barrier damage against ischemic stroke by targeting endothelin-1/Akt pathway in astrocytes
Source: Front Pharmacol. 2025 May 16;16:1571552. doi: 10.3389/fphar.2025.1571552 (PMC12122491; doi:10.3389/fphar.2025.1571552)

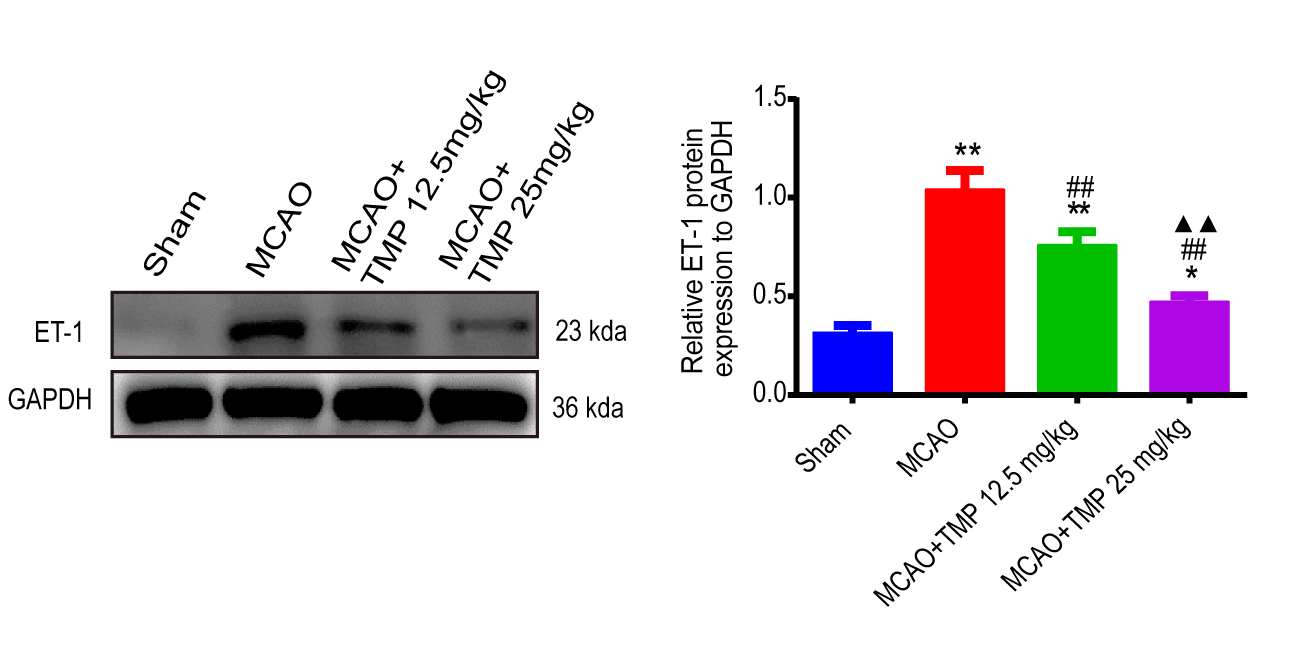

Supplement: Supplementary file 1 [file Image3.tif]

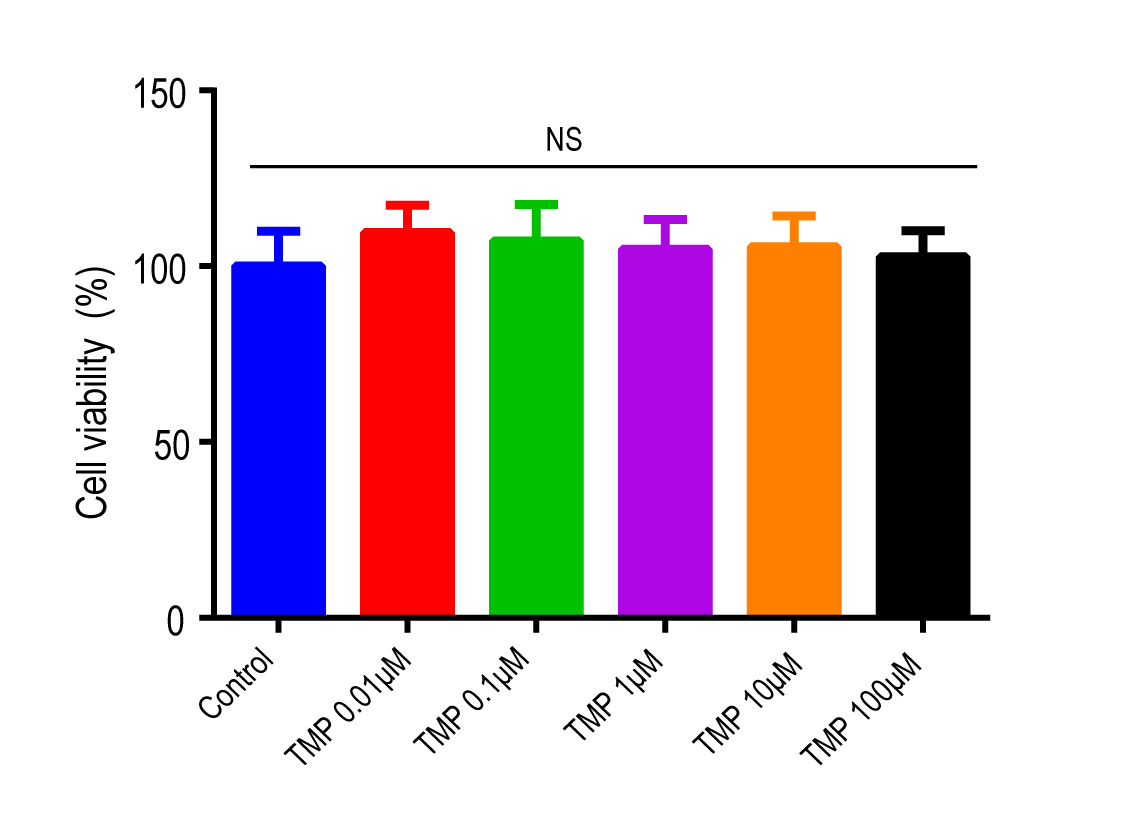

Supplement: Supplementary file 2 [file Image2.tif]

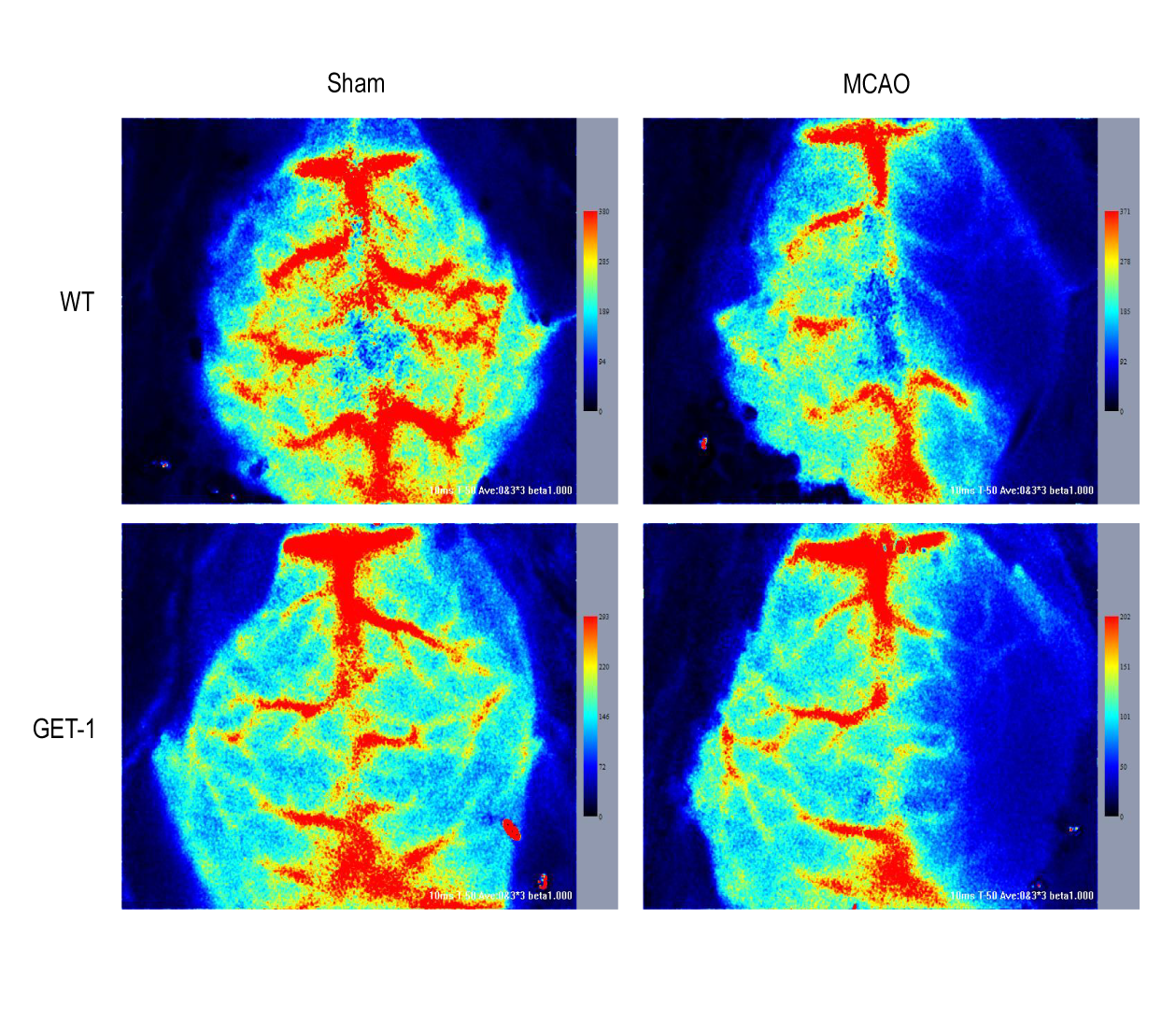

Supplement: Supplementary file 3 [file Image1.tif]
